# Using Pandemic Data to Examine the Government's Pandemic Prevention Measures and Its Support Policies for Vulnerable Groups

**DOI:** 10.3389/fpubh.2022.882872

**Published:** 2022-04-06

**Authors:** Xuefeng Li, Yaping Fang, Guoliang Chen, Zhaohui Liu

**Affiliations:** ^1^School of Economics, Zhejiang University, Hangzhou, China; ^2^The Affiliated Hospital of Southwest Medical University, Luzhou, China; ^3^School of Economics, Zhejiang University of Technology, Hangzhou, China; ^4^Guangdong Key Laboratory of Stomatology, Guanghua School of Stomatology, Hospital of Stomatology, Sun Yat-sen University, Guangdong, China

**Keywords:** COVID-19, pandemic prevention measures, vulnerable groups, WHO, Google

## Abstract

The outbreak of COVID-19 and the uncertainty it brings have created enormous pressure on governments to control the global pandemic and restore economic growth. It is an inevitable choice for governments of various countries to seek to control the pandemic and to provide support such as subsidies to people who lose their jobs or cannot work. However, governments should evaluate their pandemic policies to determine their effectiveness. To maintain social stability and help vulnerable groups, governments also must determine when subsidies are needed and when these support policies should be withdrawn. This research demonstrates that the administration of vaccines and the wearing of masks have a relatively limited impact on preventing the spread of the COVID-19 virus. By contrast, strict school closure policies combined with personal movement restrictions are more helpful in mitigating the spread of the virus. Compared with vaccine policies and wearing masks, controlling internal movement is the most effective way to manage the pandemic in schools. Additionally, economic support such as subsidies for the unemployed and underemployed is not only conducive to prevention of the virus' spread but also to economic recovery and social stability. When the pandemic is brought under control, economic support for vulnerable groups can be gradually reduced or even withdrawn.

## Introduction

Governments all over the world have responded to the COVID-19 outbreak. Many countries have chosen to develop and inject vaccines, require people to wear masks, restrict activities in public places, and close schools to control the spread of the virus. The World Health Organization (WHO) recommends immediate vaccinations, physical distancing of at least 6 feet from others, mask-wearing, and frequent hand-washing with alcohol-based hand sanitizer or soap and water to keep oneself and others safe during the pandemic. However, Johns Hopkins University's global pandemic map indicates that the prevention of new cases in various countries remains highly difficult. On February 15, 2022, the daily new cases of COVID-19 in the United States reached 163,525; although that figure represented a sharp drop from the peak of 900,000 daily cases, the situation is still not optimistic. Although the total number of vaccine doses administered has reached 10.3 billion globally, the emergence of the COVID omicron variant has resulted in 1,630,848 new cases per day, and the cumulative number of confirmed cases has reached 415 million. Dense urban areas were affected more severely in the early stages of the pandemic. However, in late 2021, COVID-19 spread to less densely populated areas of some countries as vaccinations became common in cities. There is growing evidence that poorer communities have higher mortality rates. Without adequate protection during the pandemic, many low-income workers were forced to continue working to survive, exacerbating the spread of the disease in low-income countries. The continuation of the pandemic has a lasting impact not only on people's health, lives, work, and even psychology, it also has a greater impact on vulnerable groups such as people who have lost their jobs or cannot work.

COVID-19 has directly affected people's health and has damaged human capital. Additionally, the pandemic has triggered the worst economic crisis since World War II. The continued spread of the virus has resulted in the unemployment of workers in sectors such as tourism, hotels, restaurants, and aviation. Moreover, flight bans and strict entry restrictions have created worker shortages in some countries that rely on foreign workers. Furthermore, containment and quarantine measures are damaging global supply chains and causing sharp declines in trade and foreign investment. Measures to curb the spread of the virus have hit small and medium-sized enterprises and entrepreneurs particularly hard. Some industries or businesses that can adopt remote working styles have been relatively unaffected by the pandemic, while the digital divide has exacerbated its impact in rural areas or in countries with low levels of digitalization. Face with this crisis, to support vulnerable groups such as people who have lost their jobs or cannot work, governments around the world have adopted supportive policies such as loan concessions, subsidies, and transfer payments, and have increased spending on healthcare. These measures could lead to greater government debt. To fill the fiscal gap, some governments have been forced to postpone or cancel some investment projects to save money. The decline in capital and infrastructure investments will, in turn, lead to more job losses. Therefore, controlling the pandemic and restoring economic growth are the way to break out of this vicious circle.

Political and scientific leaders are wrestling with many questions. Can vaccines alone prevent the spread of the virus? Would other measures be more effective in alleviating the pandemic's impact on the global economy, especially on vulnerable groups? How much support is needed to provide cash and other subsidies to unemployed people? When can these support policies be withdrawn? To explore these challenges and to answer these questions, we have used daily pandemic data released by WHO, government response tracking data released by Oxford University, and travel data provided by Google. Our research found that restricting people's movements is the most direct and effective means of pandemic prevention, and the use of vaccines and mask requirements is a reliable policy combination. In terms of economic support policies, countries are using measures including loans and subsidies to assist vulnerable groups, and this support can gradually be withdrawn as control over the pandemic grows.

## Literature Review

Some scholars have used WHO data to predict the trends of the pandemic or to provide reference for the formulation of strategies. Riverol (1) used WHO's COVID-19 dashboard to study Cuba's disease prevention strategies. Idogawa et al. (2) used data from Johns Hopkins University's global coronavirus dashboard, data from the European Center for Disease Prevention and Control, and WHO situation reports to construct an interactive graph of coronavirus disease cases and deaths to help track COVID-19 trends over time. Appiah and Kursah (3) used WHO data to study the impact of confirmed COVID-19 cases on the number of deaths at the global and regional levels, demonstrating a positive correlation between the two.

Additional literature has examined the role of Oxford University's COVID-19 Government Response Tracker in outbreak tracking research. Hale et al. (4) describe the database's ability to track national and local government policies and interventions. The project provides an easily accessible, near real-time resource of critical data for policymakers and researchers to understand the impact of policies on disease transmission and socioeconomic wellbeing. Wong et al. (5) examined the impact of various non-pharmaceutical interventions on local and global control of COVID-19, finding that stricter containment and control measures may lead to better control. Gani (6) analyzed the effect of COVID-19 government stringency measures on hotel occupancy rates.

Saha et al. (7) used Google's COVID-19 community mobility reports to analyze the government-imposed lockdown and its impact on community mobility in India. Research reveals that India's lockdown measures have led to a sharp drop in social mobility trends. Likewise, Zhu et al. (8) used Google community mobility reports examining social distancing in Latin America during the pandemic to assess the rigor of the government's response to COVID-19 and, based on this, to make policy recommendations for changing local courses of action. Fang et al. (9, 10) used Baidu Index to examine the China's stock markets and COVID-19.

## Data Source and Statistical Description

The COVID-19 government response tracker we used is from the University of Oxford.^1^ This data is used primarily to track and compare policy responses in different countries or regions of the world in a rigorous and consistent manner. It includes a stringency index, a containment and health index, and an economic support index. The stringency index records information on social distancing measures, coded from eight indicators: school closures, workplace closures, cancellations of public events, restrictions on the size of gatherings, closures to public transport, stay-at-home requirements, restrictions on internal movement, and international travel. The containment and health index is coded by three indicators representing public awareness campaigns, testing policies, and contact tracing. The index represents the government's emergency policies for health systems such as the coronavirus testing regime. The economic support index is composed of two indicators—government income support and household projected debt or contracted relief—and represents the government's income support policy for citizens in times of crisis. Each of these three metrics is expressed in simply summed scores of the underlying metrics, rescaled to a range from 0 to 100. These indices are used for comparative purposes and should not be interpreted as a rating of the appropriateness or effectiveness of a country's response (4). The daily number of new cases comes from the WHO. The mobility data comes from Google and is primarily used to indicate how visits to locations such as grocery stores and pharmacies, parks, transit stations, retail and recreation sites, residences, and workplaces vary by geographic area. The research sample covers 230 countries around the world, and the interval is from January 4, 2020, to December 31, 2021. A statistical description of the main variables is presented in Table 1. Taking the daily number of new cases as an example, the maximum value is 414188, the minimum value is −32952, and the average value is 1,976.

**Table 1:** Statistical description.

**Variable**	**Government response index**	**Mean**	**Sd**	**Min**	**p50**	**Max**
**GRI**		**55.20**	**15.40**	**0**	**57.30**	**91.20**
CHI	Containment health index	56.80	15.80	0	58.90	93.40
WHO	Daily new cases	1,976	9,907	−32,952	92	414,188
School	School closures	1.800	1.100	0	2	3
Move	Restrict internal movement of people	0.900	0.900	0	1	2
Vaccine	Availability of vaccines	1.600	2	0	0	5
Testing	Availability of detection	2.100	0.800	0	2	3
Information	Public information	1.800	0.500	0	2	2
Elder	Care policy for the elderly population	1.400	1.100	0	1	3
Invest	New investment in vaccines	504,955	4.500e+07	−0.100	0	7.900e+09
Ask	Requirements for wearing masks	2.500	1.200	0	3	4
Tracking	Epidemiological investigation	1.400	0.700	0	2	2
Support	The level of government cash subsidies for the unemployed	0.600	0.500	0	1	1
Retail	Retail and entertainment	−15.30	28.40	−97	−14	156
Trans it	Traffic system	−20.20	29.40	−100	−22	135
Grocery	Grocery stores and pharmacies	6.900	32.40	−98	3	228
Parks	Garden	9.600	55.80	−100	−5	670
Workplace	Workplace	−19.30	20	−92	−18	106
Resident	Place of residence	6.800	9.200	−35	6	55

## Empirical Research

In our panel regression model, the dependent variable is the number of new cases per day. The independent variables are relevant pandemic data from Oxford University, WHO, and Google. The panel regression using the above data (see Table 2) indicates, in columns (1) and (2), that the containment health index and government response index coefficients are significantly negative, indicating that the overall pandemic prevention policy has effectively reduced new cases, but the vaccine administration and mask-wearing policies are not effective. The direct containment effect of the pandemic did not appear, and the coefficients of the two were positive. This may be due to the fact that, after receiving vaccinations or wearing masks, people relaxed their vigilance against the virus and did not pay attention to maintaining social distance, et cetera, resulting in an increase in the number of new cases. However, it can be found from column (6) that the policy combination of vaccine popularization and mask-wearing can control the pandemic more effectively.

**Table 2:** The impact of pandemic control policies on new cases.

	**(1)**	**(2)**	**(3)**	**(4)**	**(5)**	**(6)**
	**WHO**	**WHO**	**WHO**	**WHO**	**WHO**	**WHO**
CHI	−19.7^**^ (8.9)					
GRI		−16.3^*^ (9.1)				
Vaccine	355.7^*^	348.1^*^	275.2	218.1^***^	267.6	513.4^***^
	(211.7)	(210.9)	(173.1)	(82.5)	(171.2)	(194.2)
Ask _	725.3^*^	715.5^*^	681.3	658.5^*^	684.2	732.3^**^
	(425.5)	(425.4)	(416.5)	(344.3)	(416.4)	(356.3)
School			−531.7^*^		−423.6	
			(300.7)		(277.5)	
Move _				−506.1^**^	−399.1^**^	−508.7^**^
				(237.7)	(191.9)	(238.9)
Vaccine # Ask _						−106.7^*^ (54.6)
Testing	−500.1	−506.6	−582.3	−585.8	−605.6	
	(855.6)	(855.3)	(879.5)	(878.4)	(885.9)	
Information	210.3	208.8	247.6	192.4	231.4	
	(294.1)	(293.5)	(310.2)	(288.2)	(306.7)	
Elder	103.2	69.0	63.5	80.0	120.0	
	(235.6)	(233.8)	(197.3)	(208.5)	(187.5)	
Invest	−0.0	−0.0	−0.0	−0.0	−0.0	
	(0.0)	(0.0)	(0.0)	(0.0)	(0.0)	
Tracking	587.3	567.6	537.6	523.4	553.6	
	(610.2)	(611.7)	(620.0)	(607.8)	(623.6)	
_cons	−202.3	−324.7	156.5	−683.1	207.6	−908.3
	(476.6)	(491.7)	(462.8)	(1034.4)	(465.8)	(1065.6)
*N*	80,833	80,833	80,833	80,833	80,833	80,833

*, **, *** *indicates statistical significance at the 10%, 5%, and 1% level*.

The calculation results in columns (3) and (4) reveal that in the control policy, the “school” and “move” variables are significantly negative, indicating that the stricter the policy of closing schools and restricting the movement of people, the better the control over the spread of the virus. However, it can be found from column (4) that if the variable controlling internal mobility is added, the school policy is not significant; that is, the improvement effect of closing schools is ultimately brought about by reducing internal mobility. From the structure of columns (5) and (6), compared with vaccine policies and wearing masks, controlling internal flow is the most effective way to limit the growth in new cases.

In response to the impact of the pandemic, governments around the world need to increase support policies such as subsidies for vulnerable groups to maintain social stability. OECD defined vulnerable and disadvantaged groups as: young people; people with a disability; minorities; migrants; aboriginals; and early school leavers. Due to the availability of data, we used the unemployed to represent vulnerable groups. The dependent variable in Table 3 is the government's cash subsidies to the unemployed. From the calculation results, column (1) reveals that with the increase of new cases, the economic support for vulnerable groups will also increase. Additionally, when economic activities gradually resume (Google data indicates an increase in retail sales, park traffic, workplace traffic, etc.), per capita economic support will also decrease accordingly.

**Table 3:** The impact of economic activity on economic support policies.

	**(1)**	**(2)**
	**Support**	**Support**
WHO	9.8^**^	3.6^***^
	(4.7)	(0.7)
Retail		−130.9^***^
		(39.3)
Parks		−794.6^**^
		(330.1)
Workplace		−2267.2*
		(1341.9)
Trans it		2260.0
		(2029.3)
Grocery		849.2
		(1232.6)
Resident		−5522.9
		(4450.9)
_cons	4341.2^***^	1188738.0^***^
	(1396.7)	(91766.8)
*N*	57769	39541

*, **, *** *indicates statistical significance at the 10%, 5%, and 1% level*.

## Conclusions and Policy Recommendations

Using data from Oxford University, WHO, and Google, this paper studies the effectiveness of government pandemic control policies and economic support policies for vulnerable groups. The results of the study reveal that, overall, the pandemic prevention policies of various countries have effectively reduced the number of new cases. The policy combination of vaccine popularization and mask-wearing can better control the spread of the virus alongside strict policies regarding school closure and the restriction of people's movements. The positive impact of school closures is attributed to reductions in mobility. When compared with policies regarding vaccines and mask-wearing, controlling people's movements is the most effective way to limit new cases. As new cases increase, so should financial support for vulnerable groups. When the pandemic is gradually brought under control, government support policies such as subsidies for vulnerable groups should be slowly reduced until they are withdrawn.

Based on the above research conclusions, we believe that, to more effectively control the spread of the COVID-19 pandemic, multiple policy combinations are needed. Chief among them are that the entire population be vaccinated, that people continue to wear masks and socially distance, and that people who test positive for COVID-19 be quarantined in time to stop the spread of the virus. These policy combinations are more effective than a single policy. After new cases are found in a specific area, the use of isolation measures to restrict people's movements is an effective means of blocking the spread of the virus. For example, when outbreaks are detected in schools and workplaces, timely closure of these sites can help curb the spread of the virus and reduce new cases. Additionally, public awareness campaigns urging vigilance against COVID-19 are effective, highlighting the importance of communication and dissemination of scientific knowledge on disease prevention among various stakeholders in social groups during a pandemic.

During a pandemic, it is necessary to adopt a subsidy policy for vulnerable groups such as people who lose their jobs or cannot work. This is invaluable for these groups to ensure basic living security, alleviate the impact of the pandemic on society, and maintain social stability. However, once the pandemic is under control, these support policies can be reduced gradually, allowing funds to be diverted to driving the economic recovery, thereby providing more employment opportunities for vulnerable groups.

## Data Availability Statement

Publicly available datasets were analyzed in this study. This data can be found at: https://ceicdata.com/.

## Author Contributions

XL: writing—original draft. YF: literature. GC: investigation and design. ZL: draft writing. All authors contributed to the article and approved the submitted version.

## Funding

The authors thank for the support by Philosophy and Social Sciences Planning Project of Zhejiang Province (18NDJC215YB); National Natural Science Foundation of China (71874160); Fundamental Research Funds for the Provincial Universities of Zhejiang (GB202002003; GB201902002); the Major Humanities and Social Sciences Research Projects in Zhejiang Province (2021QN050), Zhejiang Soft Science Research Program (2022C25015).

## Conflict of Interest

The authors declare that the research was conducted in the absence of any commercial or financial relationships that could be construed as a potential conflict of interest.

## Publisher's Note

All claims expressed in this article are solely those of the authors and do not necessarily represent those of their affiliated organizations, or those of the publisher, the editors and the reviewers. Any product that may be evaluated in this article, or claim that may be made by its manufacturer, is not guaranteed or endorsed by the publisher.

## Footnotes

^1^We got the data of COVID-19 Government Response Tracker from https://www.bsg.ox.ac.uk. The Mobility Report CSV Documentation data come from https://www.google.com/covid19/mobility/data_documentation.html?hl=en.
